# A qualitative study on self-regulated learning among high performing medical students

**DOI:** 10.1186/s12909-021-02712-w

**Published:** 2021-06-05

**Authors:** Chan Choong Foong, Nur Liyana Bashir Ghouse, An Jie Lye, Nurul Atira Khairul Anhar Holder, Vinod Pallath, Wei-Han Hong, Joong Hiong Sim, Jamuna Vadivelu

**Affiliations:** Medical Education and Research Development Unit (MERDU), Faculty of Medicine, University of Malaya, 50603 Kuala Lumpur, Malaysia

**Keywords:** Self-regulated learning, Undergraduate, High performing medical students, Pre-clinical, Effective learning, Characteristics, Qualitative research

## Abstract

**Background:**

Self-regulated learning (SRL) is an important contributing element to the academic success of students. Literature suggests that the understanding of SRL among medical students is obscure as there is still some uncertainty about whether high performing medical students use SRL. This study explored the characteristics of high performing medical students from the SRL perspective to gain a better understanding of the application of SRL for effective learning.

**Methods:**

Twenty-one students who scored at the 90th percentile in written knowledge-based assessment consented to participate in this study. Each student wrote a guided reflective journal and subsequently attended a semi-structured interview. Students were prompted to explain the rationales for their answers. The data were then analysed using thematic analysis to identify patterns among these students from the SRL perspective. Two coders analysed the data independently and discussed the codes to reach a consensus.

**Results:**

High performing students set goals, made plans, and motivated themselves to achieve the goals. They put consistent efforts into their studies and applied effective learning strategies. They also employed coping mechanisms to deal with challenges. High performing students regularly evaluated their performance and adopted new strategies.

**Conclusions:**

This study reported that high performing students applied SRL and described the rationales of practice. Medical schools could design SRL-driven interventions to enhance the learning experiences of medical students. Recommendations are made for students on how to apply SRL.

**Supplementary Information:**

The online version contains supplementary material available at 10.1186/s12909-021-02712-w.

## Background

Accomplishing a medical degree requires a substantial amount of time, energy, and resources for medical schools and their students. Constant effort by medical educators is to explore elements leading to academic success among their students. How medical students regulate their learning is believed to be vital to academic success [1–4]. Students practise self-regulated learning (SRL) when they take responsibility for their learning to achieve goals [5, 6]. SRL is demonstrated in three cyclical phases, which are the forethought, performance, and self-reflection phases [7]. In the forethought phase, students set goals and plan their strategies upon analysing and assessing their abilities [7]. Their motivation beliefs would influence the standard of goal setting [7, 8]. In the performance phase, they implement the planned strategies and monitor their progress to ensure that they remain focused on achieving their goals [7]. In the self-reflection phase, students evaluate the effectiveness of the implemented strategies and adopt appropriate measures by reviewing the outcomes [7]. The three phases are cyclical as actions taken by students in each phase would influence their actions in the subsequent phase [5, 6]. An example of a broken cycle is whereby students do not set a goal, resulting in their strategies being directionless, resulting in students losing their focus easily [8, 9].

SRL is essential for higher education because adult learners are granted greater autonomy and responsibility for their learning. As adults, they are expected to take initiative in managing their studies [10]. In the field of medical education, SRL possibly explains differences in academic achievement between high performing and low performing students. First, self-efficacy is positively associated with examination grades [11]. Students with better examination grades show higher task value and self-efficacy [1]. The motivational belief and emotions of students are significant contributors to examination grades [2]. A SRL study in a clinical setting showed high performing students had used strategic thinking and actions during a clinical task [12]. Similarly, there is a positive correlation between the application of SRL and clinical skills development [13]. This positive association between SRL and academic achievement is supported in a scoping review [14]. It is noted that low performing students fail to apply SRL in overcoming academic failures [15]. SRL also influences the well-being of students. The application of SRL was reported to be negatively associated with depression among medical students [16]. Low performing students showed greater anxiety and frustration [2]. It was further elaborated where medical students with inadequate use of SRL expressed negative emotions [15]. In addition, low performing medical students demonstrated a lack of metacognitive knowledge to assess their abilities [17].

In contrast to the supporting evidence, some studies argue that there is no significant relationship between the application of SRL by medical students and their acquisition of knowledge [1, 18]. A previous study suggested that the application of SRL was limited to reflection and monitoring when describing its relationship with the acquisition of knowledge [19]. Another study highlighted that there were no differences in prior knowledge, non-verbal reasoning, metacognitive monitoring, or self-efficacy between high performing and low performing students before they enrolled in the medical programme [20]. Overall, there is a weak correlation between SRL and academic achievement among medical students [21].

The aforementioned literature suggests that the understanding of SRL among medical students is obscure as there is still some uncertainty about whether high performing medical students use SRL. To contribute a clearer insight, the present study aimed to answer the following question: what were the characteristics of high performing students from the SRL perspective?

It is important to investigate how medical students regulate their studies and analyse why these regulating behaviours are adopted if medical schools desire to develop a theory-based intervention in enhancing students’ learning experiences [3]. A previous study has proposed to compare different approaches of using SRL among high performing students to understand the elements for effective learning [22]. Comparisons between high and low performing students on how they learn would help differentiate the effective and ineffective measures for learning [20, 23]. A previous study explored how and why some low performing students failed from the SRL perspective and made recommendations for interventions [15]. Only by comparing why medical students have failed or succeeded, the SRL-driven intervention could inform the good and bad practices of students.

## Methods

The study was carried out at the University of Malaya, a public-funded institution of higher education in Malaysia. Spread across 5-years, the medical curriculum is vertically integrated [24]. Year 1 and Year 2 pre-clinical students have clinical immersion experiences (e.g. history taking and physical examination, procedural skills) before entering into Year 3, Year 4, and Year 5 where clinical clerkship at the teaching hospital takes place. The pre-clinical curriculum is horizontally integrated [24] where the learning content is divided into system blocks (e.g. musculoskeletal sciences, cardiovascular sciences, haematology). The common teaching and learning activities are lectures, interactive and multi-disciplines seminars, problem-based learning sessions, laboratory sessions, and clinical days.

### Participants

At the end of the academic year of 2018/2019, high performing students from Year 1 and Year 2 were identified based on their knowledge-based written assessment results. Students who scored at the 90th percentile of an assessment are generally recognised as high performing students [25]. Thirty-one students fulfilled the selection criteria. At the beginning of the academic year of 2019/2020, all students were invited to a meeting with one of the researchers, NAKAH. The researcher had no prior personal relationships with the students. In the meeting, the students were informed about the aim, procedures, and ethics of the study. They were also invited to attend an interview as well as to complete a guided reflective journal. The students were notified that their participation in this study was voluntary, and anonymity was assured. Upon agreeing to participate in this study, the students completed the consent form and an interview was scheduled. A total of 21 students completed the guided reflective journal but only 14 students attended the interview as scheduled. Important dates (e.g. interview dates, dates of receiving the guided reflective journals) were documented.

### Data collection

An empty template of the guided reflective journal was emailed to all students who consented to participate in the study. The guided reflective journal was initially developed for low performing students to describe their experience according to Gibbs’ cycle [26]. Terms were modified to suit the context of high performing students (e.g. “failed” was changed to “passed”). An additional file shows questions asked in the guided reflective journal in more detail (see Additional file 1).

The students completed the guided reflective journals as a take-home task during their free time. They were given two weeks to submit the journals. It was aimed to provide sufficient time for students to recall their previous experiences before writing their reflective journals.

Once the guided reflective journals were received, a semi-structured interview was conducted individually with the students by NAKAH. The interviewer read the guided reflective journal of the students, familiarised herself with their background, and left notes on what to seek for further description during the interviews. The interviewer tried to build a good rapport with them at the beginning of the interviews. Some interview questions were listed below:

- When did you realise that you would score well in the exam?
- How did you feel after receiving the exam results?
- What did you do before/during/after attending the teaching activities? Why did you do so?
- If you could start the academic year again, which strategy would you maintain? Why?

Students were prompted to give rationales for their actions or strategies used. In cases where the given information was insufficient, the interviewer followed up the question with contingency questions to obtain a more informative response:

- How did you write your notes?
- Why did you say that understanding is important?
- Why did you change the strategy?
- Why did you ensure that you always listen to the lecturers and stay awake during the lectures?

Each one-to-one interview lasted about 45 to 70 min and was recorded with a digital recorder, with verbal consent from the students. Students were told that they could inform the interviewer if they felt uncomfortable to express further on an interview question. The interviews were transcribed verbatim.

### Data analysis

The data consists of interview transcripts and guided reflective journals. Thematic analysis was used to analyse the characteristics of the students from the SRL perspective. Thematic analysis applies a flexible and functional approach that provides rich and detailed findings [27]. It is a technique used in qualitative analysis for identifying, analysing, and developing a theme or pattern to answer research questions [27]. In further detail, this approach is classified into 6 steps which are: familiarising oneself with the data, generating initial codes, searching for themes, reviewing themes, defining and naming themes, and producing the report [27].

The interview transcripts were read multiple times to gain familiarity. The researchers, FCC and NLBG coded the transcripts and guided reflective journals for the Year 1 students independently. Coders reminded themselves that they should not be rigid with the data to match the data according to the SRL perspective. Thematic analysis can be either a deductive or inductive analysis, though the analysis is guided by a pre-determined framework [28]. Once all data were coded, discussions were held between the two coders to compare their independently coded data and resolve any discrepancies. The two coders subsequently coded Year 2 students’ transcripts and guided reflective journals using the agreed codes for Year 1 students. Then, all codes were inspected whether they described both Year 1 and Year 2 students, or it only applied to a particular year of studies. No additional code was observed in the data from Year 2 students. Last, the codes were grouped into themes and were named based on the SRL perspective.

## Results

Five themes emerged from the thematic analysis describing the characteristics of high performing students from SRL perspective: (a) Goal setting, planning, and sources of motivation, (b) Consistency in studies, (c) Using effective learning strategies, (d) Coping mechanism when facing challenges, and (e) Evaluation of performance, reactions, and adaptations. Student quotes were given pseudonyms to preserve anonymity. The quotes might contain some grammatical errors but were uncorrected as the intact phrases deliver the tacit meaning of how non-native students communicate.

### Goal setting, planning, and sources of motivation

#### Goal setting

Students specified their goals that they must “pass the exam” (IS16; IS21) and “didn’t want to fail” (IS1) to be able to “graduate on time” (JS20). Some students targeted to achieve “as high as I could” (IS2), and hereby in the case where the target was not achieved, at least they passed the exam (IS12, Quote 1, refer to Table 1). Some students rationalised the importance of setting goals (IS9, Quote 2).

**Table 1:** Quotes from interview transcripts (IS) and guided reflective journals (JS)

Sources	Quote No.	Quotes
IS12	1	“Instead of setting an objective to pass the exam, I will set a higher objective, like scoring an A or a distinction in the exam. If things did not turn out (as) how I wish, at least I can pass (the exam). Yeah, I will always set my objective higher than (what is) necessary.”
IS9	2	“Knowing what you want to do as in if you want to pass the exam, and then you need to know how to do it. If you want to pass the exam, then (you should) study. If you know what you want, then you (should) know how to do [obtain] it.”
IS12	3	“Planning is the only way for me to eliminate the feeling of insecurity and nervousness when (it) comes to studies.”
IS4	4	“If I don’t plan, I won’t know if I am behind or ahead (of goals).”
IS3	5	“I feel like I know everything during the study week, I can answer any questions from my friends (and) I don’t need to think further (of it when I answer). When I do past year (questions) and do questions (given by) seniors. I can say [answer] straight away without needing to revise the answers, so I think I can do well (in the exam)”
JS17	6	“Having the kind of thoughts that patients might suffer more if I’m incompetent in my future career has always prompted me to learn as much and as fast as I can and I will feel bad and guilty for not studying”
JS13	7	“I would say that what motivate me (the) most are my own expectations and the desire to make my parents proud. Before every exam, I would set a certain goal and push myself hard to achieve it. As for my parents, I believed that they have given me the best and this is my way to repay them.”
IS8	8	“Because if I have the passion, then I will be disciplined to support my passion. If I don’t have the passion, I will not have the desire to be disciplined since I don’t care what I am doing. So, passion is the source that drives (motivates) other attitudes, such as discipline and hardworking.”
IS2	9	“I realised if I just memorised and didn’t understand it, I wouldn’t be able to explain when a patient came to ask me ‘why this (happened)’ because I just memorised it. That’s why I need to understand it so (that) I can give a better care (to patients)”
IS1	10	“I read (lecture notes) to know what we are going to study, like (having) a general view of what we are going to study that day. So, I don’t enter the class not [without] knowing what I am going to study that day.”
IS8	11	“If you don’t get enough sleep, you will feel (that) you have a lot of negative emotions the next day, and the negative emotions will make us less efficient and enthusiastic to do (things)”
IS16	12	“Because some doctors will give very good explanations and some explanations are also not easily found in textbooks. For me, when the doctor explains it, it is easier to remember because some of the explanations are very simple and easy to understand.”
IS17	13	“I will read up on the topics in textbooks and make my own notes by compiling the points from lecture notes, textbooks and articles based on the learning objectives given in lecture notes, which I personally think, are quite concise and easy for me to revise before clinical sessions and exams.”
JS8	14	“Since I have learned bit by bit throughout the year, it doesn’t take me much hard work during the study week to cram all the knowledge and when I am consistent, I feel more confident with my ability which creates a virtuous circle for me to work harder and fare better.”
IS16	15	“If you understand the concept, you can explain (it) in detail and relate that topic to another topic.”
JS7	16	“Understanding (the content) will prepare you for a real clinical setting because it [theory and practice] are not the same. With the understanding, you will be able to apply (the knowledge) in order to solve a problem.”
JS11	17	“(By) understanding things and not blindly memorising, (would) keep the information longer in our brain.”
JS12	18	“...I find (that) it is easier for me to understand something when I re-illustrate it in my own words and diagrams, and that is when (a) mind map comes into play. Flow charts are the one(s) that I love when illustrating pathophysiology of a disease, as it shows me the whole process of how a healthy person can eventually fall sick, together with the signs and symptoms presented. Hence, it is clearer [easier] for me to point out the principles behind each investigation and the management that I should prescribe to target either the symptoms or the causes.”
IS4	19	“Sometimes you have to know your priorities, like which one has to go first, which one can hold on.”
IS16	20	“...Because if I delayed it to next week, I might have forgotten what the doctors said during lectures as I said some (points) I might not have taken it (them) right. There are a few (points) that might not (be) important but just refreshing my memory. And if I were to postpone it to next week, it would delay my schedule for the next week as well.”
IS21	21	“...you need to make yourself think that me [I am] being appreciated by myself...If you keep studying without giving yourself any rest, then you will feel burned out at the end. You will (wonder) why I am suffering so much, why I am doing this? So, rewarding yourself is very important.”
IS21	22	“...I look back and realise I have spent too much time on this topic and missed other topics, then I just go back to (read) the lecture note that hasn't been ticked by me. After I finish (a topic), then I will put a tick, it is effective for me because it will guide you whether you miss a lot of lectures or you cover all the lectures...”
IS20	23	“…sometimes they want to talk to me (but) I will say ‘later, after class’”
JS19	24	“Every weekend, we usually plan to try (out food at) a new restaurant. We also spend one of the days playing bowling and badminton. These have helped me to relieve stress and be able (to) carry out a healthy lifestyle at the same time.”
IS1	25	“Because it is better to ask the lecturers than remembering something in a wrong way and not understanding what I am learning. For me, it’s important to understand what I am studying. So, I usually email to further understand or confirm that I am remembering it in the right way.”
IS4	26	“Because I feel I cannot do it on my own, sometimes it is very saturated or very difficult. Sometimes they [my friends] might not have remembered whatever I remember, so I can just help them, sometimes whatever they remembered I don’t. So, when we discuss we get the best of both worlds.”
IS3	27	“[Muslim]... I trust in His power and the power of ‘dua’ [prayer of supplication]. There are miracles and everything because when we pray it’s like a new spirit to open your heart. It’s a new vibe if we pray, there is (a) positive vibe for me and easy for me to concentrate...”
IS2	28	“[Sikh]...I always believe that two things will lead me to succeed in life which are prayers and hard work. Nothing beats those two.”
JS12	29	“[Christian]...If you believe in God or guarding angels, then it would be easier for you when dealing with problems…they know what I am experiencing, listen to all my wishes, and ‘help’ me when things become complicated, and that is what I call it as miracle.”
IS1	30	“Do the weekly questions…and sometimes do the USMLE questions for that block…For me that helps me determine what I know and what I don’t. It helps me see how much I know…I do those questions to help me apply my knowledge better and know where I stand in my knowledge, like how much knowledge I have on that block.”
IS15	31	“I did some sample questions…it was from there that I found out which topic that I wasn’t strong [good] at, and I would study more on that topic.”
IS4	32	“...if you score well in your midterm assessment, that means you understand your foundation (module) properly.”
IS2	33	“As transitioning from module 1 to module 2, I was trying to adapt to find a study method that will suit me...and when I saw my study methods are working based on the post-midterm assessment results...I was quite comfortable for the rest of my Year 1.”
IS5	34	“…I only studied during weekends…no need to do daily study… It was because of the wrong method. One week before the midterm assessment, I wasn’t able to cover everything and I broke down, I felt like I couldn’t do it…I changed and started studying every day and from 8 to 10 and 10 to 12 (midnight), then weekends and try to cover as much I can.”
IS2	35	“I had a problem of coping at first due to the transition…I coped with the overload by being more organised and systematic in planning what I would study for that particular day and how long I would take to cover it.”
IS4	36	“Because (when) I feel it is not working, why (do I have to) waste my time? So, I try to find another method with the hope that it will increase my productivity. If I don’t try it, then I am not going to get anything; if I try, there is a chance that I might be better. So, I will take that chance.”
IS12	37	“...part of it comes from doctors’ and your friends’ compliments...I feel satisfied when I can explain something to them and solve their problems when they are asking questions to me and I can answer their questions...it’s that satisfaction that pushes me all along the way. When you did something well then (and) receive compliments from others, it builds up your confidence and you feel that you want to have that satisfaction all the time, so you will keep performing.”

#### Plan

Once goals were set, it was common for students to plan what they were going to do to achieve the goals in their studies. They planned “what I should do today, for a week ahead, for a month ahead” (IS9) for their studies to be “systematic and organised” (IS2). For instance, to identify the types of cases and patients the day before clerking patients at wards, or to download lecture notes the day before the lectures were conducted. Plans were kept in “phone calendar and hardcopy calendar in the room” (IS5). To illustrate, plan-making was a sense of security for students (IS12, Quote 3; IS4 Quote 4).

#### Sources of motivation

To achieve a satisfying academic achievement, students had different sources of extrinsic motivation. Some students believed in their abilities to acquire medical content despite being told that pursuing a medical degree was difficult. They were unaffected by the “negativities” (JS3) and instead, they were determined to “prove people wrong” (IS5). Students were “confident” (IS2; IS21) that they would pass the examinations. This is because they believed that they were well prepared for the examinations (IS3, Quote 5).

Some students were motivated in becoming good doctors. Upon graduation, they wish to be “approachable, trustworthy, considerate, caring, and competent” doctors (JS17). They must study hard as “someone’s life will be in my hands” (IS17), or they would feel guilty (JS17, Quote 6).

Parents were seen as a “strong motivating factor” (JS2) and “sources of strength” (JS7) for some students. They “desired to make (their) parents proud” (JS13). By excelling in their studies, some students thought it would “make them [their parents] think they have done a good job as parents” (JS5). Students hoped they could reciprocate the love of their parents (JS13, Quote 7).

On the other hand, some students were instinctively inclined towards medicine as it “intrigued” (JS6) them. Learning medicine is fun, fascinating, and enjoyable. They felt that “medicine is interesting, full of mysteries and has a lot of discoveries to be made” (JS5), hence motivating these students to be hardworking in their studies (IS8, Quote 8). In addition, sources of motivation might influence the goal setting and strategic choices of the students. To answer queries of patients, students recognised the need to understand the content instead of rote-memorising it (IS2, Quote 9).

### Consistency in studies

#### Before attending the classes

Lecture notes were available to students before the classes. Students downloaded and at least skimmed and scanned the lecture notes before attending classes. Some students defined the need of doing to obtain a general view of what is going to learn (IS1, Quote 10).

#### During the classes

Students made sure they had “sufficient sleep” (JS8) before attending classes, and they attended almost all classes organised by the faculty. During the classes, they “concentrated” (JS9; IS21), “paid attention” (IS2; JS11), and “listened attentively” (JS1; IS3) to the content delivered by lecturers. They also “jotted down the important notes and explanations” (JS20). Students justified the need of having sufficient sleep and paying attention during the classes (IS8, Quote 11; IS16, Quote 12).

#### After attending the classes

After attending the lectures, students revised the content of the classes. They “read the lecture notes again and tried to understand everything written in the notes” (JS8). Some students believed that it would be more productive for them as their memory was still “fresh on that day” (JS11).

When confusion arose, students sought clarifications by “looking up additional references, materials in books and the internet to help (them) better understand a particular topic” (JS2). Students used either YouTube or Google/internet search as these sources “explained it in a simple manner” (IS1).

It was also common that students made their notes based on their understanding, which was “a more simplified version compared to the lecture notes” (JS9). Some students also clarified their process of making these notes. In their opinion, note-making was essential in assisting them to “organise the information and making it easier to review later especially during study week” (JS4; IS17, Quote 13).

When students were prompted for the main key to their high performance, “consistency” (IS21; JS11; JS13; JS12) was repeatedly mentioned. The tips for high performance were studying “every day” (IS4; JS3; JS2; JS16) and being “disciplined” (IS6; J20; JS8; JS9). Students clarified the necessity to perform routine revisions (JS8, Quote 14). In addition, students “did early preparation” (IS21) one or two months before the final examination. For instance, they revised what had been learnt months ago.

### Using effective learning strategies

#### Associations and meaning making

Students made associations with content from lectures, rather than merely memorising the facts. For instance, they understood the content by “attempting to understand the theories and reasoning behind a topic” (JS17; JS16) and “actively thinking about the significance of the materials studied” (JS8). This helped to relate the particular content to other content, apply the knowledge and store the knowledge as long-term memory (IS16, Quote 15; JS7, Quote 16; JS11, Quote 17).

#### Mind maps

Students frequently used imagery, such as mind maps and flowcharts to visualise the associations. The imagery helped them to “connect the dots” (IS1) and it was “easier for them to understand” (JS12). For instance, they summarised the points in their own words and made them into mind maps or flowcharts while reading through the notes (JS12, Quote 18).

#### Time management

Apart from understanding the content and generating them into mind maps, it was common for students to manage their time well by avoiding “procrastination” (JS20; IS18; IS17) or “dragging their tasks” (JS12; IS9). They further explained the rationales by mentioning that they might have forgotten some points and felt insecure if they kept on delaying their tasks. Moreover, some students prioritised certain tasks by judging on the task value and urgency to finish the tasks on time. Prioritisation was important as understanding one topic might be a prerequisite or connected to other content (IS4, Quote 19; IS16, Quote 20).

#### Self-rewards

Some students gave themselves rewards “after reaching the target for that period” (JS9) based on their “to-do list” (JS17). For instance, they rewarded themselves by spending some time on their social life and hobbies or rewarding themselves with some good food after completing their tasks. They perceived that self-rewards made them feel appreciated by themselves and avoided feeling burned out from their studies (IS21, Quote 21).

#### Self-records

While applying the learning strategies aforementioned, students recorded their task progress by “writing down what they have finished or haven’t finished” (IS1). For instance, some students included information, such as the deadline and the number of attempts they had made to revise a particular topic in the records. By putting a remark (e.g. “a tick”), they were able to know how much time they had spent on a particular topic and eventually served as a guide for them (IS21, Quote 22).

#### Environment

Students used different ways to create conducive surroundings for their studies. Some students minimised phone usage during lectures (IS3; IS9); some students had water or snacks to stay awake (IS21; IS12) or asked their friends to wake them up (JS15; IS21); some students neither talked to their friends nor allowed their friends to chat loudly or disturb them (IS3; IS17; IS20, Quote 23).

### Coping mechanisms when facing challenges

#### De-stress

It seemed that life as a medical student was not always smooth. Students also took personal measures to cope with the pressure they faced in medical school. They highlighted the importance of “taking breaks” (IS1; JS4; JS19) and “relaxing” (JS5; IS16; IS18) during their study periods. For instance, some students “allocated time for exercise, hobbies and/or entertainment” (JS8; IS18) before continuing to study. Students further explained the necessity of doing so to manage their stress (JS19, Quote 24).

#### Help-seeking

Apart from self-coping, when high performing students faced difficulties in their studies, they acknowledged their limitations and sought help from others. They solved the confusion by “asking the lecturers” (IS3; IS2; IS18) or by “having a discussion with their peers” (IS4; JS17; IS16). Whereas, when students needed emotional support, students often turned to their parents and friends for advice and encouragement. For instance, some students would “call their family members and friends every day to make sure that they were not alone” (IS3). Students also justified their actions to seek help from lecturers and peers (IS1, Quote 25; IS4, Quote 26).

#### Religious support

Religious support was also stated by students as an enabler for high performance. Students with different religions mentioned prayers were helpful as they believed that prayers helped them form positive vibes and it was easier for them to concentrate on their studies after praying (IS3, Quote 27; IS2, Quote 28; JS12, Quote 29).

### Evaluation of performance, adaptations, and reactions

#### Evaluation

To evaluate the effectiveness of their learning strategies, students implemented several methods. Students did some practice by answering questions from past examinations, USMLE, seniors, or from the internet. Some students also “explained to myself [themselves]” (IS12; IS4) or “explained the ideas to other people to test my [their] understanding” (IS8). Students rationalised the importance of assessing their understanding after they revised the medical content. If the outcome were unsatisfied, they would relearn the content (IS1, Quote 30; IS15, Quote 31).

Based on their midterm assessment result, students evaluated their level of understanding and decided whether to continue or modify their learning strategies until they find the strategies that work best for them (IS4, Quote 32; IS2, Quote 33).

#### Adaptations

High performing students were willing to modify their learning strategies upon recognising that previous strategies were less effective. They re-thought the applied strategies when they encountered difficulties in their studies. They decided to “change” (IS2; IS20) or did “trial and error” (IS21; IS18; JS20) with different strategies until they identified strategies that were “more suitable” (JS20; IS9) for them (IS5, Quote 34; IS2, Quote 35). Students recognised that they were “struggling” (JS1; IS4) and they hoped they could remember, understand or perform “better” in the future (JS21; IS18; IS4, Quote 36).

#### Reactions

They felt “happy” (IS8; IS21; IS18), “satisfied and excited” (IS12), either when they received satisfying results, compliments, or when they were able to answer the questions in discussions with lecturers or peers. They further explained their feelings by mentioning that their “hard work (had) paid off” (IS2) and the satisfaction pushed them along the way as well as “building up their confidence” (IS12, Quote 37).

## Discussion

**Fig. 1:**
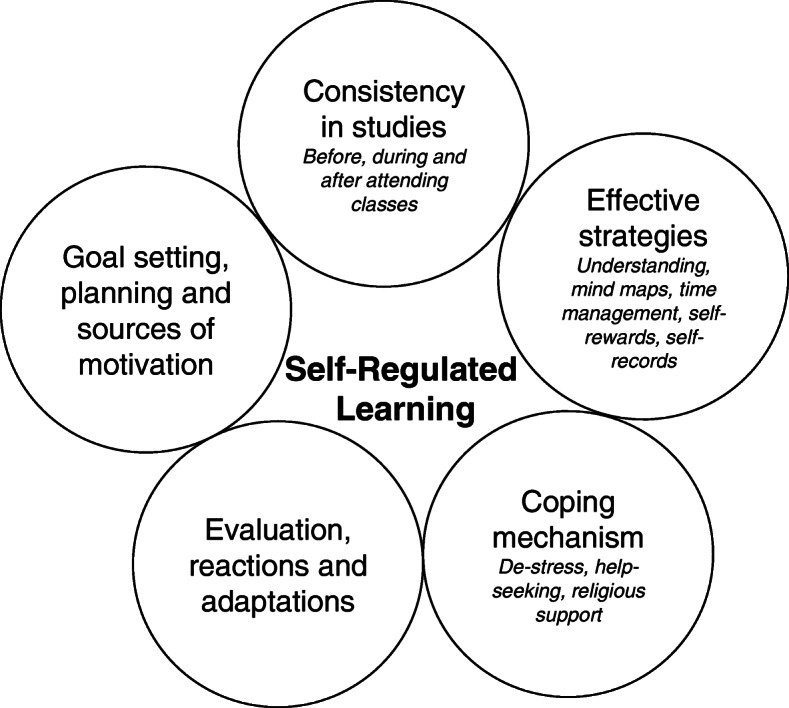
A possible mechanism of application of SRL among high performing students

To the best knowledge of the authors, the present study is the first qualitative study investigating the application of SRL among higher performing students. The present findings provided empirical evidence to support the theoretical application of SRL (Fig. 1), which was lacking in the literature. The evidence is important as it consolidates the learning theory in explaining effective and ineffective learning among medical students. In the discussion, past findings involving high and low performing medical students were compared with the present findings to further verify the distinctive application of SRL among high performing students.

In the forethought phase, there is a difference in goal setting and motivation between high performing and low performing medical students. Low performing students tend to operate with a minimal and short-term goal (i.e. passing the exam) [15] but high performing students tend to operate with a higher and long-term goal. To illustrate the differences, despite setting “passing the exam” as the bottom-line of their academic achievements, high performing students would still aim for a higher target. The present findings are consistent with the study of Abraham and colleagues, which mentioned that high performing students were determined to achieve better academic achievement [29]. In addition, high performing students were highly motivated as compared to low performing students [1]. Motivation is an important element of learning because it predicts academic success [30].

In the performance phase, differences between high performing and low performing students were shown in the learning strategies, consistency in studies, and coping mechanisms. In terms of the learning strategies, low performing students tended to just rote-memorise the medical content [15], while high performing students tended to study by associations and meaning making. In addition, low performing students viewed the content taught in each lecture as disparities and did not see them in a holistic manner [31]. High performing students rationalised the need for understanding to apply the medical content and improve their memory retention. Likewise, they believed that understanding the medical content (i.e. physiological concepts) in their first year of studies would eventually help them to understand other subjects in the subsequent year of studies, and to build their foundation knowledge for future clinical practices [29]. It was also identified that high performing students seemed to apply learning strategies more effectively. For instance, they organised the medical content to ease their understanding by using mind maps and good time management for their studies. In contrast, low performing students demonstrated poor organisational skills [31–33], where they did not organise the information well and showed poor time management for their studies [31, 32, 34].

Another difference between high performing and low performing medical students was identified in the consistency in studies. In the present study, high performing students emphasised the need to study consistently before, during, and after attending classes. In contrast, low performing students had poor class attendance [11, 34]. Lack of preparation and effort was a possible reason for low performing students to have failed their studies [9, 34]. High performing students had higher self-efficacy during examinations as they were more prepared, whereas low performing students demonstrated lower self-efficacy and higher anxiety during examinations that might have affected their academic achievement [1, 2, 21].

In addition, high performing medical students applied better coping mechanisms when facing challenges than low performing students did. Based on the present findings, high performing students indicated a more positive attitude towards help-seeking. They were willing to acknowledge their limitations in acquiring the medical content and tried to seek formal support from lecturers. In reverse, low performing students were reluctant to seek formal support as they did not want to be identified as “problematic students” [15]. Furthermore, high performing students also sought help from their peers by having group discussions to clarify their doubts in their studies [29]. Whereas low performing students refused to seek help from their peers as they considered them as “competitors”[15]. It should also be noted that low performing students often did not have peers who were socially and academically supportive towards them [31, 35]. Surprisingly, high performing students mentioned the role of religiosity as a part of the coping mechanism. A possible explanation is that college students with stronger religiosity tend to spend more hours in studies and fewer hours in social activities [36]. This relationship between religiosity and academic success was discussed in a general context [37] but it may require further investigations to enrich findings of religious support among medical students.

In the self-reflection phase, high performing students were willing to take the initiative in changing their learning strategies after the strategies were proved less effective by referring to their academic achievements. On the other hand, low performing students tended to face their failures by normalising them as they believed that other students also faced the same struggle as they did [15]. Moreover, they put the blame on external attributes, such as having an unfair exam or a mean examiner, rather than self-reflecting [15]. Low performing students were considered to be more dependent on the guidance given by teachers and they were less confident in managing their studies [29]. Apart from having a passive attitude, the problem-solving skills of low performing students seemed to be linear and simplified, instead of simultaneous and addressing the root cause and they showed a lack of reflection in their behaviours [31]. By considering these findings, it may infer that a positive and active attitude pertaining to failure is an essential element leading to academic success.

Another difference found in the self-reflection phase showed that high performing medical students were adaptive to change their strategies upon reflecting on the effectiveness. They did “trial and error” until identifying strategies that suited them. Low performing students were unadaptable to change and remained the same strategies [9]. Nevertheless, some low performing students did try to change their strategies but they simply made changes with the hope of “trial and success” without evaluating the strategies beforehand [31]. Low performing students seldom practised reflections [19]. Without proper reflections, they might repeat the same mistakes in their studies.

### Implications for future practice and study

Based on the findings and discussions, the application of SRL is discussed for future practices in medical schools and students. Medical schools are recommended to promote SRL to students, both the theory and its applications [4]. Workshops could be conducted at the early stage of the programme for students to begin applying SRL [38, 39]. While students are progressing through the programme, the schools could encourage students to attempt and overcome challenging tasks, as a platform for them to experiment with their strategies and to build self-efficacy [40]. The schools could teach students using a structured SRL framework in practising the forethought, performance, and self-reflection phases [9]. It is not advisable for medical schools to assume that medical students are primarily high-functioning and successful [2, 41]. The schools should not solely expect all students to practise effective SRL by themselves [14]. As SRL is not the only theory for learning enhancement, future studies could explore how other learning theories could be applied for medical students.

It is hoped that medical schools could improve the motivation of students, promote goal setting and self-efficacy of students in the forethought phase [1, 11]. Medical students ought to recognise their future responsibilities, as they should be accountable for patients’ safety. Students should be assisted to nurture a professional identity on how they foresee themselves as future doctors and the kind of doctors they want to become (e.g. approachable, trustworthy, considerate, caring). Medical schools could help medical students in developing intrinsic motivation because their motivation is mouldable [42]. Professional identity formation enables the growth of motivation [43]. Therefore, medical schools need to incorporate instructional strategies aiding the formation of professional identities since the beginning of the programme [44]. Narrative reflection may be a useful tool in forming professional identities. It could be used by medical students to tell about their journey and provide assessable meanings of the medical journeys [44]. As some high performing students were extrinsically motivated, it might be worthwhile for future studies to explore how students could internalise their extrinsic motivation.

Moving on to the performance phase, students are encouraged to attend and concentrate in classes as they could have missed some important content [11, 34]. High performing students used tactics (e.g. candies, reminder) for them to remain focused in their studies. For the after-class revisions, they are advised to understand the medical content instead of rote-memorising it. Future studies should be undertaken to answer the remaining questions, such as the necessity and appropriateness for students to memorise facts (e.g. anatomical content) and the contents that they should not learn by memorising. In addition, it is necessary for students to be aware of their learning needs and apply suitable strategies to attain their goals such as using mind maps, self-records, and self-rewards [14]. Whenever they are struggling, they should recognise their limitations and actively seek formal and informal support. Meanwhile, the schools are suggested to intervene with students proactively [41]. Students should not perceive help-seeking as a disgrace or a weakness [45] whereas the schools should not rely on students to take initiative [41].

Self-reflection is essential to complete the SRL cycle. Students should regularly evaluate the gap between their actual performance and goals. Apart from referring to official examination results, lecturers or peers could provide feedback during classes [12]. Medical schools are proposed to design formative assessments and provide opportunities for students to practise reflections [17]. Medical students should reflect on their limitations on both mental and cognitive aspects [19, 46]. For those disappointing experiences, it is advisable for students to be more open-minded and adaptive to changes. In addition, students should not blame their failures on external attributes [15]. Schools could design a structured worksheet to support students in monitoring their performances and self-record their adaptive approaches [47]. Meanwhile, lecturers should portray themselves as role models for students and share their experience of using SRL [12]. Reflection is an important practice but it is a challenge for students to have accurate self-knowledge and find a balance between stringency and leniency to assess their performance [48, 49].

### Strengths and limitations of the study

Interview data were triangulated with guided reflective journals of the students, and two coders analysed the data independently. These methods were applied to enhance the credibility of the study [50]. Description of the medical curriculum clarified the transferability of the findings [50].

Limitations of the study were identified. First, this was a self-reported study, and students were the only data source for the study. Future studies may interview lecturers and peers to enrich the data from different perspectives. It is unclear whether the self-perception of students would vary from the perceptions of lecturers and peers [15]. Second, high performing students were defined as those who performed well in knowledge-based examinations based on the grading system of the institution. The application of SRL among high performing students in clinical examinations was not explored. Third, the present study investigated 21 high performing students, and the findings were compared with characteristics of low performing students reported in past qualitative [15, 29, 32, 33] and quantitative studies [1, 2, 11, 19, 21, 34]. Nonetheless, future studies could investigate both high and low performing students in the same institution to minimise possible interferences due to different curriculum, pedagogy, assessments, student support systems, and institutional culture. Last, the present study does not demonstrate the cause-effect relationship between the application of SRL and the high performance of the students, though characteristics of high performing students were reported from the SRL perspective. Future mixed-methods research (e.g. structural equation modelling) would be useful to examine this relationship.

## Conclusions

The present qualitative study explored the application of SRL among high performing students developing characteristic approaches in learning. The students applied SRL in their studies and described the rationales of their actions. Recommendations for medical schools and students in employing SRL were suggested based on the comparison between high and low performing medical students.

## Supplementary information

Additional file 1.  Guided reflective journal. The guided reflective journal was used for high performing students to describe their learning experiences. 

## Data Availability

The datasets collected and analysed during this study are available from the corresponding author on reasonable request.

## Abbreviations

SRL: Self-regulated learning
