# Supplementary material for: A qualitative study on self-regulated learning among high performing medical students
Source: BMC Med Educ. 2021 Jun 5;21:320. doi: 10.1186/s12909-021-02712-w (PMC8178823; doi:10.1186/s12909-021-02712-w)
Supplement: Supplementary file 1 — Guided reflective journal. The guided reflective journal was used for high performing students to describe their learning experiences. [file 12909_2021_2712_MOESM1_ESM.docx]

**Additional file 1**

**Guided reflective journal**

1. Personal Profile
2. Name, date of birth, age, unique or special things about yourself, previous academic experiences, and achievements before entering the university.
3. Family background, hometown, parents’ occupation, parents’ academic background, siblings, family members in the medical profession.
4. Reasons for Studying Medicine
5. Why did you choose to study medicine?
6. Did you have any pre-existing knowledge about the medical profession? If yes, what was the source of this information?
7. You had an excellent pre-university result. Why did you not consider choosing other programmes?
8. What motivated you to continue your studies?
9. What kind of doctor do you want to be?
10. Reflection

a) i. Which study approach did you use from the start until the end of the academic year?

ii. Did you encounter any problem using this study approach? How did you cope with the problem?

iii. Elaborate on your study style:

- How did you prepare for lectures/teaching sessions?
- What was your attendance (%) for lectures/teaching sessions?
- Describe your attention/behaviour during lectures/teaching sessions.
- What did you do to revise the content after lectures/teaching sessions?
- How did you manage your studies and social activities on weekends?

b) i. How was your social life from the start until the end of the academic year?

ii. Did you encounter any problem in your social life? How did you cope with this problem?

c) i. How was your personal life/family relationship from the start until the end of the academic year?

ii. Did you encounter any problem in your social life? How did you cope with this problem?

d) Is there anything else that you would like to share?

e) i. Would you consider any of the stated study approaches/styles to have contributed to your academic success? Please justify your answer.

ii. What did you think and feel about this study approach/style? Why?

iii. What have you learned from that situation?

iv. Why do you think that situation happened?

v. If you were given a chance to change your action, what would you do?

vi. Based on what happened, what is your plan for the current academic year? Describe.

vii. How will you make sure that you carry out your action plan properly? Describe.
